# Immune response in peripheral axons delays disease progression in SOD1^G93A^ mice

**DOI:** 10.1186/s12974-016-0732-2

**Published:** 2016-10-07

**Authors:** Giovanni Nardo, Maria Chiara Trolese, Giuseppe de Vito, Roberta Cecchi, Nilo Riva, Giorgia Dina, Paul R. Heath, Angelo Quattrini, Pamela J. Shaw, Vincenzo Piazza, Caterina Bendotti

**Affiliations:** 1 Laboratory of Molecular Neurobiology, Department of Neuroscience, IRCCS - Istituto di Ricerche Farmacologiche Mario Negri, Via La Masa 19, 20156 Milan, Italy; 2 NEST, Scuola Normale Superiore, Piazza San Silvestro 12, I-56127 Pisa, Italy; 3 Center for Nanotechnology Innovation @NEST, Istituto Italiano di Tecnologia, Piazza San Silvestro 12, I-56127 Pisa, Italy; 4 Neuropathology Unit, Department of Neurology, INSPE, San Raffaele Scientific Institute, Dibit II, Via Olgettina 48, 20132 Milan, Italy; 5 Department of Neuroscience, Academic Neurology Unit, Faculty of Medicine, Dentistry and Health, Sheffield Institute for Translational Neuroscience, University of Sheffield, 385 Glossop Road, Sheffield, S10 2HQ UK

**Keywords:** Amyotrophic lateral sclerosis, SOD1^G93A^ mice, Immune system, Peripheral nervous system

## Abstract

**Background:**

Increasing evidence suggests that the immune system has a beneficial role in the progression of amyotrophic lateral sclerosis (ALS) although the mechanism remains unclear. Recently, we demonstrated that motor neurons (MNs) of C57SOD1^G93A^ mice with slow disease progression activate molecules classically involved in the cross-talk with the immune system. This happens a lot less in 129SvSOD1^G93A^ mice which, while expressing the same amount of transgene, had faster disease progression and earlier axonal damage. The present study investigated whether and how the immune response is involved in the preservation of motor axons in the mouse model of familial ALS with a more benign disease course.

**Methods:**

First, the extent of axonal damage, Schwann cell proliferation, and neuromuscular junction (NMJ) denervation were compared between the two ALS mouse models at the disease onset. Then, we compared the expression levels of different immune molecules, the morphology of myelin sheaths, and the presence of blood-derived immune cell infiltrates in the sciatic nerve of the two SOD1G93A mouse strains using immunohistochemical, immunoblot, quantitative reverse transcription PCR, and rotating-polarization Coherent Anti-Stokes Raman Scattering techniques.

**Results:**

Muscle denervation, axonal dysregulation, and myelin disruption together with reduced Schwann cell proliferation are prominent in 129SvSOD1^G93A^ compared to C57SOD1^G93A^ mice at the disease onset, and this correlates with a faster disease progression in the first strain. On the contrary, a striking increase of immune molecules such as CCL2, MHCI, and C3 was seen in sciatic nerves of slow progressor C57SOD1^G93A^ mice and this was accompanied by heavy infiltration of CD8^+^ T lymphocytes and macrophages. These phenomena were not detectable in the peripheral nervous system of fast-progressing mice.

**Conclusions:**

These data show for the first time that damaged MNs in SOD1-related ALS actively recruit immune cells in the peripheral nervous system to delay muscle denervation and prolong the lifespan. On the contrary, the lack of this response has a negative impact on the disease course.

**Electronic supplementary material:**

The online version of this article (doi:10.1186/s12974-016-0732-2) contains supplementary material, which is available to authorized users.

## Introduction

Amyotrophic lateral sclerosis (ALS) is the most common neuromuscular disorder, affecting individuals from all ethnic backgrounds, with an incidence of 2–3 cases per 100,000 population per year [1]. Terminal axonal degeneration with neuromuscular junction (NMJ) denervation is an early component of motor neuron (MN) disease in ALS [2]. In addition, it has now become clear that ALS is a non-cell autonomous disease involving activation of microglia and astrocytes in the CNS [3] and infiltrates of peripheral T cells and macrophages in the spinal cord and sciatic nerve [4, 5]. These non-neuronal cells play an active role in MN death. In fact, selective ablation of mutant SOD1 in astrocytes and microglial cells and neonatal bone marrow (BM) transplantation significantly increased MN survival and lifespan [3] of mutant SOD1 transgenic mice. In contrast, T cell deficiency accelerated the disease progression in these mouse models [4, 6]. These studies have been mainly focused on the CNS, while the contribution of immune cells in the peripheral nervous system (PNS) has not been fully analyzed.

ALS is a clinically heterogeneous disease with a high variability in the rate of symptom progression even in the familial cases [7, 8]. Growing evidence indicates that also in mutant SOD1 mice, the most widely used model of familial ALS, the severity of the disease is markedly influenced by their genetic background [9–12]. In fact, we have recently obtained and characterized two mouse strains (C57BL6 and 129Sv) carrying the same copy number of the human SOD1^G93A^ transgene that showed remarked difference in the speed of symptom progression [13, 14]. In particular, the fast-progressing mice (129SvSOD1^G93A^) compared to the slow progressors (C57SOD1^G93A^) showed an earlier impairment of motor function and shorten survival, which was unrelated to the extent of MN loss in the lumbar spinal cord, despite significant differences in their MN transcriptome [9, 13, 14]. Noteworthy is the fact that rapidly progressing mice, unlike the slow progressor, showed markedly decreased expression of transcripts linked to axonal transport and neuronal projection [9, 14] at disease onset, suggesting major involvement of the axonal compartment both at the central and peripheral levels to determine the different mice phenotypes. This was then demonstrated by the ex vivo diffusion tensor imaging and histopathological analysis showing an earlier axonal degeneration in the white matter of the spinal cord of fast- compared to the slow-progressor mice [15]. In contrast, MNs of slow- but not fast-progressing mice specifically activated and translocated to the peripheral motor axons immune molecules (i.e., major histocompatibility complex I (MHCI)) directly involved in the induction of an adaptive immune response [9, 14].

In the present study, we further extended these observations analyzing in more details the involvement of the PNS and the associated immune system in the modulation of disease progression. We clearly demonstrated that the dysregulation of PNS is the origin of the difference between the two transgenic mice strains and that immune cell infiltration in this compartment is essential to delay muscle denervation of SOD1^G93A^ mice.

## Methods

### Mice

Female transgenic SOD1^G93A^ mice on C57BL/6JOlaHsd or 129SvHsd genetic background, hereafter indicated as C57SOD1^G93A^ and 129SvSOD1^G93A^, respectively, and corresponding non-transgenic (Ntg) littermates were used. Procedures involving animals and their care were conducted in conformity with institutional guidelines that comply with national (Legislative Degree 26, March, 2014) and international (EEC Council Directive 2010/63, August, 2013) laws and policies.

Animals studies were approved by the Mario Negri Institute Animal Care and Use Committee and by the Italian Ministerial decree no. 84-2013.

### Laser-captured microdissection and microarray data analysis

We extrapolated data for *β2m*, *Tap1*, *H2*_*K-D*, and *Lmp7* messenger RNA (mRNA) levels in C57-Ntg; 129Sv-Ntg, and C57-SOD1G93A laser-captured MNs from microarray analysis; the detailed procedure has been previously described in Nardo et al. [14]. Additional information is supplied in the Additional file 1.

### Immunohistochemistry

The spinal cord and sciatic nerve were processed as previously described [8]. Briefly, the mice were perfused with Tyrode’s buffer, followed by Lana’s fixative (4 % formalin and 0.4 % picric acid in 0.16 M PBS, pH 7.2) at 20 °C. The lumbar spinal cord and sciatic nerves were quickly dissected out. The tissue was left in the same fixative for 90–180 min or overnight at 4 °C, rinsed, and stored 24 h in 10 % sucrose with 0.1 % sodium azide in 0.01 M PSB at 4 °C for cryoprotection, before mounting in optimal cutting temperature compound (OCT). The spinal cords and sciatic nerves were cut, respectively, in 30- and 14-μm sections. The following primary antibodies and staining were used: rat anti-MHC class I ER-HR 52 clone (1:100; Abcam), rat anti-CCL2 (1:50; Abcam), rabbit anti-β2m (1:500; Proteintech), rabbit anti-Lmp7 (1:500; AbD Serotec), mouse anti-SMI-31 (1:1000; Sternberger Inc), rabbit anti-CD8 (1:50; Abcam), rabbit anti S100β (1:400; Sigma-Aldrich), α**-**btx (5 μg/ml) conjugated with Alexa-594 (Invitrogen), and NeuroTrace conjugated with Alexa-488 or Alexa-594 (1:500; Invitrogen). Secondary antibodies were as follows: Alexa 488 or Alexa 594 goat anti-rat, Alexa 488 or Alexa 594 goat anti-rabbit, and Alexa 594 goat anti-mouse (Invitrogen). All immunohistochemistry followed an indirect immunostaining protocol whereas peroxidase-diaminobenzidine (DAB) reaction was used for detecting MHCI in the spinal cord and DAB (brown) plus DAB-NICHEL (blue) reactions in the sciatic nerve for the double labeling of MHCI (1:00) and CD8 (1:50).

### Immunohistochemical evaluation of Lmp7 in human obturator nerve

All procedures in studies involving human participants were in accordance with the ethical standards of the institutional and/or national research committee and with the 1964 declaration of Helsinki and amendments, or comparable ethical standards. We studied a motor nerve sample from a sporadic ALS male patient, whose anterior branch of the obturator nerve had been previously biopsied for diagnostic purposes, as described [16]. This patient developed progressive lower-limb weakness at the age of 52; motor nerve biopsy led to a neuropathological diagnosis of motor neuron disease. Subsequently, he developed upper motor neuron signs, allowing a clinical diagnosis of definite ALS [17]. A normal nerve sample belonging to a 65-year-old patient with a final diagnosis of distal sensorimotor peripheral neuropathy was studied as a control. Although the histopathological analysis showed a normal nerve at time of biopsy, clinical worsening and neurophysiological follow-up subsequently allowed a final diagnosis of peripheral neuropathy. Neuropathological diagnosis was based on previous diagnostic criteria [18]. Transverse 10-μm sections of the frozen nerve were cut with a cryostat and transferred to 0.1 % poly-d-lysine-coated glass slides. The cryosections were immunostained with affinity-purified fluorescein isothiocyanate (FITC)-conjugated goat antibodies to human LMP7 (1:100; Southern Biotechnology Assoc.) and tetramethylrhodamine (TRITC) conjugated goat antibodies (Southern Biotechnology Assoc.) to human neurofilament 200 kDa (rabbit) (1:1000; Chemicon).

### Coherent Anti-Stokes Raman Scattering (CARS) analysis of the sciatic nerves

The animals were sacrificed with cervical dislocation, and their sciatic nerves were rapidly surgically explanted and frozen in dry ice in a WillCo dish (GWSt-3522; WillCo Wells, Amsterdam, The Netherlands). The samples were then thawed by immersion into warm (~38 °C) Dulbecco’s phosphate buffer saline (D8537; Sigma-Aldrich, Saint Luis, Missouri, U.S.A.) and immobilized with a custom-made electrophysiology-type anchor. The explanted sciatic nerves from C57SOD1^G93A^, 129SvSOD1^G93A^, and relative NTG mice were observed with an RP-CARS microscope [19] tuned to CH_2_ molecular vibrations yielding the average in-plane orientation anisotropy (*α*) of the chosen molecular vibrations and their average orientation (*φ*) within the sub-micrometric excitation volume [20]. The acquired z-stacks were analyzed with a custom-made Python software (Python Software Foundation, Beaverton, OR, USA) to threshold the myelin walls and to extract the stack distribution of the *α* values (we employed the same algorithm described in [21], but computed the threshold on the *α* value images). The *α* value distributions are shown in Additional file 1: Figure S1. Finally, the same z-stacks were analyzed with a second custom-made Python software to extract the stack-based average dispersion (*β* value) of the myelin-associated *φ* values over a chosen spatial scale (see Additional file 1). The *β* value is an indicator of the average micrometric longitudinal straightness of the myelin sheaths.

### Muscle denervation

Tibialis anterior muscles were dissected out and snap-frozen in isopentane cooled in liquid nitrogen. Twenty-micrometer serial longitudinal cryosections were collected on poly-lysine objective slides (VWR International). The sections were stained with anti-synaptophysin (SYP) (1:100; Synaptic System) as pre-synaptic marker. The secondary antibody was FITC-labeled goat anti-rabbit (1:500, Invitrogen). Subsequently, the sections were stained with α-bungarotoxin (α-BTX) coupled to Alexa Fluor 594 (1:1200; Invitrogen) as post synaptic marker. Fluorescence images along the *z*-axis were taken by Olympus confocal microscopy using a ×20 objective, and z-stacking was done using Fiji software (with Z-Stack Projection/SUM command) (ImageJ, US National Institutes of Health, Bethesda, MD, USA). End plates were scored as innervated if there was complete overlap between SYP and a-BTX staining and scored as denervated if they showed only α-bungarotoxin staining.

### Western blot

The mice were decapitated, and the spinal cord and sciatic nerve were rapidly dissected. The spinal cord was processed as previously described [14]. The sciatic nerves and muscles were powdered in liquid nitrogen then homogenized by sonication in ice-cold homogenization buffer centrifuged at 13,000 rpm for 15 min at 4 °C, and the supernatants were collected and stored at −80 °C. Equal amounts of total protein homogenates were loaded on polyacrylamide gels and electroblotted onto PVDF membrane (Millipore) as previously described [14]. Densitometric analysis was performed with Progenesis PG240 v2006 software (Nonlinear Dynamics). Immunoreactivity (IR) was normalized to β-tubulin, β-actin, or the total amount of protein detected by red Ponceau (Sigma-Aldrich) or SYPRO Ruby Blot (Thermo Fisher scientific). Additional information is supplied in the Additional file 1.

### Quantitative reverse transcription PCR

The spinal cords, sciatic nerves, and muscles were perfused in 0.1 M PBS, freshly collected and then frozen on dry ice. The total RNA from spinal cord was extracted using the Trizol method (Invitrogen) and purified with PureLink RNA columns (Life Technologies). For fibrous tissues (sciatic nerve and muscles), the RNeasy® Mini Kit (Qiagen) was used. The RNA samples were treated with DNase I, and reverse transcription was done with a High Capacity cDNA Reverse Transcription Kit (Life Technologies). For quantitative reverse transcription PCR (RT-qPCR), we used the TaqMan Gene expression assay (Applied Biosystems) following the manufacturer’s instructions, on complementary DNA (cDNA) specimens in triplicate, using 1X Universal PCR master mix (Life technologies) and 1X mix containing specific receptor probes. The levels of transcripts were normalized to β-actin. The mean values of the triplicate results for each animal were used as individual data for 2^−ΔΔCt^ statistical analysis. Additional information is supplied in the Additional file 1.

### Statistical analysis

The Mann-Whitney test was used to compare differences between two groups and one-way or two-way ANOVA was used to compare differences between more than two groups. For the CARS analysis, z-stack-based indicators (i.e., the *α* values and the *β* values) were statistically analyzed with a general linear mixed model implemented in R (R Foundation, Vienna, Austria), using the strain (C57 vs. 129Sv) and the mutation (Ntg vs. G93A) as fixed factors and the nerve as a random factor. Post hoc comparisons between the experimental groups were instead performed with Mann-Whitney test on the specimen-based medians.

## Results

### The severity of disease in SOD1^G93A^ mice correlates with earlier muscle denervation

**Fig. 1:**
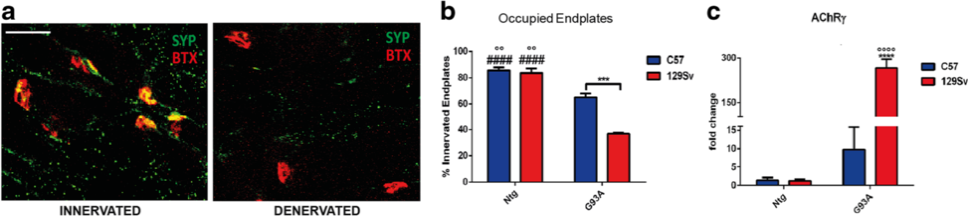
Muscle innervation is more impaired in fast- (129SvSOD1^G93A^) than in slow-progressing C57SOD1^G93A^ mice. **a, b** Denervation was analyzed in tibialis anterior muscle of 129SvSOD1^G93A^ and C57OD1^G93A^ strains and Ntg littermates. **a** α-Bungarotoxin (*BTX*, *red*) was used to identify the postsynaptic domain; synaptophysin (*SYP*, *green*) was used to identify pre-synaptic terminals (*scale bar*, 50 μm). **b** Mean ± SEM (*n* = 3 mice; ~100 bungarotoxin-positive endplates per animal were randomly chosen and analyzed). ***P value <0.001 (C57G93A vs 129SvG93A); °°*P* value <0.01 (129SvNtg and C57Ntg vs C57G93A); ^*####*^ *P* value <0.0001 (129SvNtg and C57Ntg vs 129SvG93A) by two-way ANOVA with Tukey’s post-analysis. **c** Real-time PCR for AChR-γ transcript in the TA muscles of C57SOD1^G93A^ and 129SvSOD1^G93A^ mice compared with the Ntg littermates. Data are normalized to β-actin and expressed as the mean (*±*SEM)-fold change ratio between the C57SOD1^G93A^ mice, 129SvSOD1^G93A^ mice, and controls (*n* = 4 per group). ****P value <0.0001 (C57G93A vs 129SvG93A); *°°°°P* value <0.0001 (129SvG93A vs NtgC57 and Ntg129Sv); by two-way ANOVA with Tukey’s post-analysis

The present study indicates that muscle innervation at the disease onset is more severely perturbed in 129SvSOD1^G93A^ than in C57SOD1^G93A^ mice confirming the prediction of a faster disease progression in the former mice as compared to the second ones slow progressor. Denervation of the tibialis anterior (TA) muscle was more extensive in the former mice, with only 37 ± 1.5 % of occupied end plates compared to 65 ± 5 % in the C57SOD1^G93A^ mice (Fig. 1a, b). In addition, the transcript levels of the gamma subunit of the cholinergic nicotinic receptor (AChR-γ), the expression of which is abundant in the presence of denervated or defective NMJs [22], showed significantly greater upregulation in the TA muscles of the fast-progressing mice compared to the slow progressors (Fig. 1c).

### Axonal dysregulation and reduced Schwann cell proliferation are prominent in fast-progressing mice at disease onset

**Fig. 2:**
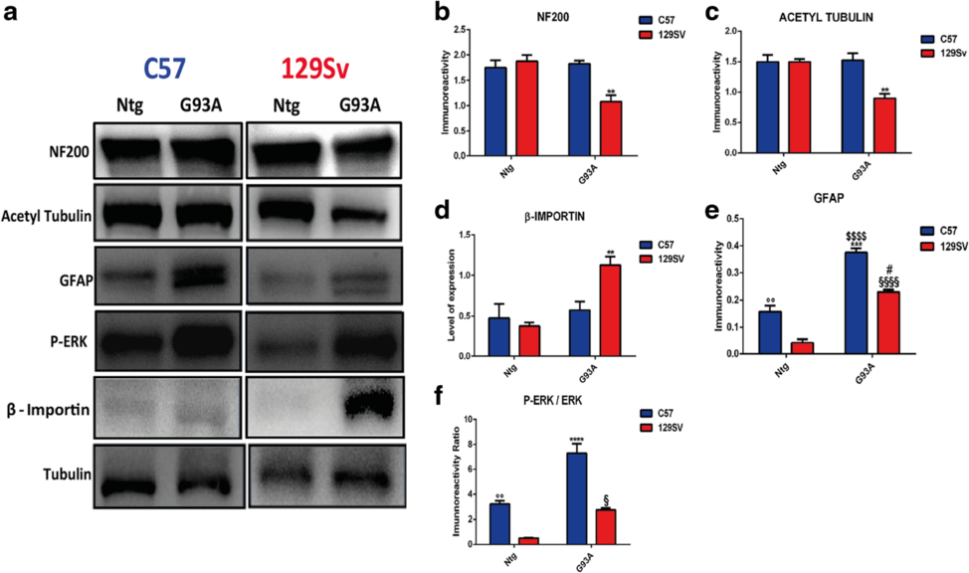
Sciatic nerve dysregulation of 129SvSOD1^G93A^ mice at disease onset. **a** Representative western blots images of neurofilaments, acetylated tubulin, GFAP, P-ERK, and β-importin, and tubulin on sciatic nerve extracts from the C57SOD1^G93A^, 129SvSOD1^G93A^, and Ntg littermates at the onset of the disease. **b**–**f** Densitometric analysis shows reductions in the expression of **b** neurofilaments and **c** acetyl tubulin as well as specific activation of **d** β-importin in the sciatic nerve of the 129SvSOD1^G93A^ mice. In addition, activation of **e** GFAP and **f** P-ERK was reduced in the sciatic nerve of the 129SvNtg and 129SvSOD1^G93A^ mice. P-ERK levels were normalized to relative levels of ERK (not shown). Mean ± SEM (*n* = 4 per group). **P value <0.01; ***P value <0.001; ****P value <0.0001 (C57G93A vs 129SvG93A in **b**–**f**); *°°P* value <0.001; (NtgC57 vs Ntg129Sv in **e** and **f**); ^*#*^ *P* value <0.05 (129SvG93A vs NtgC57 in **e**); ^*$$$$*^ *P* value <0.0001 (C57G93A vs NtgC57 and Ntg129Sv in **e**); ^*§*^ *P* value <0.05; ^*§§§§*^ *P* value <0.0001 (129SvG93A vs NtgC57 and Ntg129Sv in **e**); (129SvG93A vs Ntg129Sv in **f**) by two-way ANOVA with Tukey’s post-analysis

The sciatic nerves of the fast-progressing mice showed a marked downregulation of neurofilaments (Fig. 2a, b) and acetylated tubulin (Fig. 2a, c) and upregulation of β*-*importin (Fig. 2a, d), three reliable indices of axonal stress and degeneration [23–25]. This effect was not seen in the slow-progressing mice at the same disease stage. In addition, there were differences in Schwann cells (SCs) between the two ALS mouse strains. In fact, levels of glial fibrillary acidic protein (GFAP) (Fig. 2a, e) and phospho (P)-ERK (Fig. 2a, f), which are known to induce de-differentiation and proliferation of the SCs during axonal regeneration [26, 27], were higher in the slow-progressing mice than in those with fast disease progression. Such differences reflect the different basal levels of the proteins in the two non-transgenic strains, being higher in C57Ntg than 129SvNtg mice. This is also evident when the two mouse strains were analyzed at the same age (98 days) (Additional file 1: Figure S2a–c). Thus, even though both SOD1^G93A^ mouse strains can activate SC de-differentiation and proliferation in the sciatic nerves, in terms of absolute levels, this appears more pronounced in mice with a slow disease course compared to the fast progressors.

**Fig. 3:**
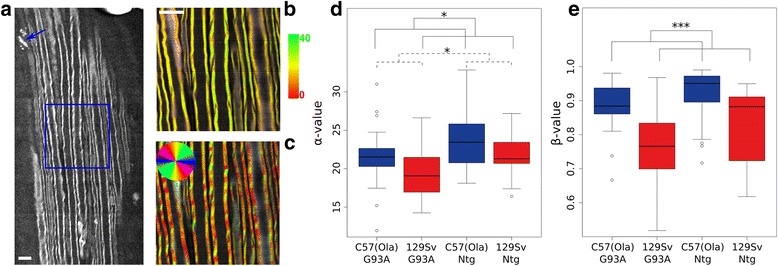
Myelin is more disorganized in sciatic nerves of 129Sv-SOD1^G93A^ at disease onset. **a** Large-scale CARS image of a longitudinal optical section of a 129Sv-SOD1^G93A^ mouse sciatic nerve. Myelin debris reminiscent of Wallerian axonal degeneration are indicated by a *blue arrow*. **b** Magnification of the *blue square* area in the *panel a* representative of anisotropy RP-CARS image highlighting the *α* value mapped onto the hue according to the color bar. **c** Bond-orientation RP-CARS image of the same area depicted in **b** mapping the *φ* value onto the hue. The saturation and the value of the images in **a** and **b** were constructed as previously described [15]. *Scale bar*, 10 μm. On average, 2.9 z-stacks, each composed of 10–11 slices (200 × 200 pixels per slice, corresponding to an area of 50 μm × 50 μm on the sample) were acquired for each nerve, and when possible, the sampling was performed at the proximal, the central, and the distal sections of the nerve. For all the mice except one, we observed both sciatic nerves. **d**, **e** Box plots showing the *α* value (**d**) and the *β* value (**e**) in the different conditions. *P value <0.05; ***P value <0.001 by general linear mixed model

Based on these results, RP-CARS microscope analysis was exploited to detect the myelin-associated *α* values in the sciatic nerve of slow- and fast-progressing mice. *α* value is an indicator of the myelin molecular order, and it is tightly associated with the myelin health status [19, 20, 28]. Representative RP-CARS images of sciatic nerve longitudinal optical sections are shown in Fig. 3: a large-scale traditional-CARS image (Fig. 3a) and two images obtained by color-mapping the *α* (Fig. 3b) and the *φ* (Fig. 3c) values are represented. The statistical analysis performed on the explanted sciatic nerves from ten C57 mice (five Ntg; five SOD1^G93A^) and eight 129Sv mice (3 Ntg; 5 SOD1^G93A^), revealed that the 129Sv strain is associated with a decrease of 1.9 with respect to the C57 strain in the *α* value (*P* < 0.05), and the presence of the G93A mutation is associated with a decrease of 2.0 with respect to the Ntg (*P* < 0.05) in the same indicator. Box plot depicting the *α* values in the different conditions is shown in Fig. 3d. The same microscopic acquisitions were also used for the analysis of the average dispersion of the myelin-associated *φ* values. This indicator, called *β* value, reflects the longitudinal straightness of the myelin sheaths over a chosen spatial scale (see Additional file 1). The statistical analysis of the results highlighted a significant decrease (*P* < 0.001) in the *β* value for the 129Sv compared to the C57 strain at a spatial scale of 5 μm (Fig. 3e). We noted that factor-of-100 changes (from 0.5 to 45 μm) in the spatial scale do not significantly alter the results implying that the differences occur over a broad range of spatial scales. It should be pointed out, however, that the observed C57 mice were 1 month older than the 129Sv mice and that the resultant length value is known to correlate with age in wild-type mice. Nonetheless, the difference (around 0.1) observed here between mice of different strains (slow- and fast-progressing mice, 98 vs. 135 days) is about 30 times larger than the levels previously observed for 5-month-older mice belonging to the same strain (28 vs. 140 days) [29]. In addition, post hoc analysis between experimental groups revealed that the difference in the *β* value between the C57 and the 129Sv strains is significant in the SOD1G93A mice (*P* < 0.01) and not significant in the Ntg case (*P* ≈ 0.13).

### Sustained activation of the MHCI pathway, centrally and peripherally, occurs in slow- but not fast-progressing mice

We have previously demonstrated that MHCI is activated more in MNs of slow-progressing than fast-progressing mice at disease onset [14]. The present study extended the analysis to other components for MHCI presentation such as *Lmp7*, *H2_D-K*, *Tap1*, and *β2m.* Basal mRNA levels of *H2_D-K* and *β2m* were significantly higher in the C57Ntg than 129SvNtg MNs, with fold changes, respectively, of 3.6 and 4.7 times (Additional file 1: Figure S3a). When we measured the mRNA expression of *Lmp7*, *H2_D-K, Tap1*, and *β2m* obtained by microarray analysis of laser-captured MNs from the C57SOD1^G93A^ mice (*n* = 4) compared to the Ntg littermates (*n* = 4) at the presymptomatic (56 days), symptom onset (135 days), and symptomatic (147 days) stages of the disease, all four genes showed similar patterns of expression during disease progression, with a peak at disease onset and a progressive decrease at the symptomatic stage (Additional file 1: Figure S3b). No differences were found for the 129Sv mice [14]. For this reason, we examined the expression and distribution of the components of this pathway in more detail in the CNS and PNS of the C57SOD1^G93A^ mice.

**Fig. 4:**
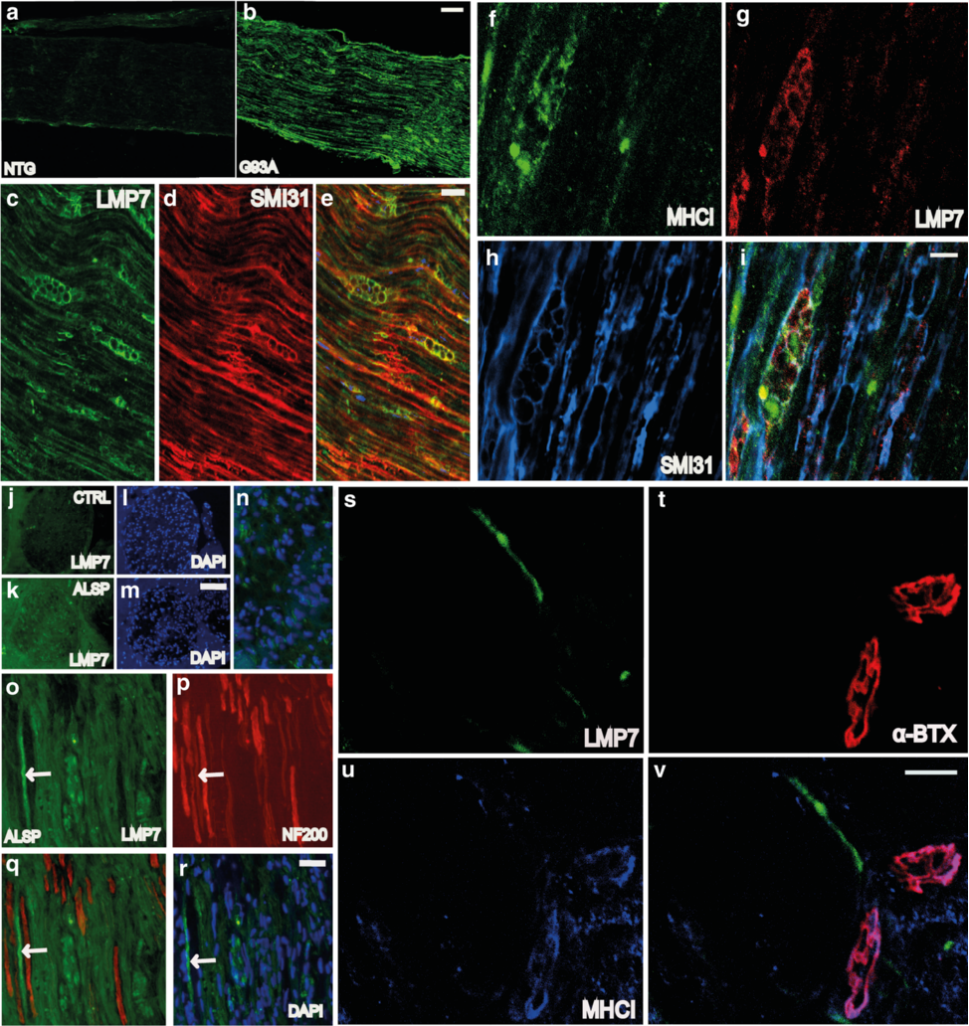
LMP7 is activated in the peripheral nerve axons of C57SOD1^G93A^ mice and ALS patients. **a, b** LMP7 immunoreactivity (*green*) is strikingly upregulated in the sciatic nerve of the C57SOD1^G93A^ mice at the disease onset (G93A) compared to the Ntg littermates (NTG); *scale bar*, 100 μm. **c**–**e** Representative co-localization of LMP7 with SMI-31 (axonal marker; *red*) in the sciatic nerve of C57SOD1^G93A^ mice; scale bar, 20 μm. **f**–**i** MHCI (*green*), LMP7 (*red*), and SMI-31 (*blue*) co-localization in the sciatic nerve of C57SOD1^G93A^ mice, highlighting MHCI-LMP7 co-immunoreactivity in motor axons with vacuoles typical of Wallerian degeneration. **j**–**n** Transverse section of an obturator nerve biopsy from a sporadic ALS patient (ALSP) and control (CTRL), showing a marked LMP7 activation (*green*) in ALS patient; DAPI: *blue*; scale bar, 50 μm; scale bar, 150 μm. **o**–**r** Longitudinal section of the obturator nerve biopsy from an ALS patient, showing LMP7 (*green*) co-localization with neurofilaments (*red*); scale bar: 50 μm; **s**–**v** LMP7 is expressed by terminal motor axons but not at the NMJ of C57SOD1G93A mice; LMP7 (*green*), α-BTX (*red*), and MHCI (*blue*); scale bar, 50 μm

**Fig. 5:**
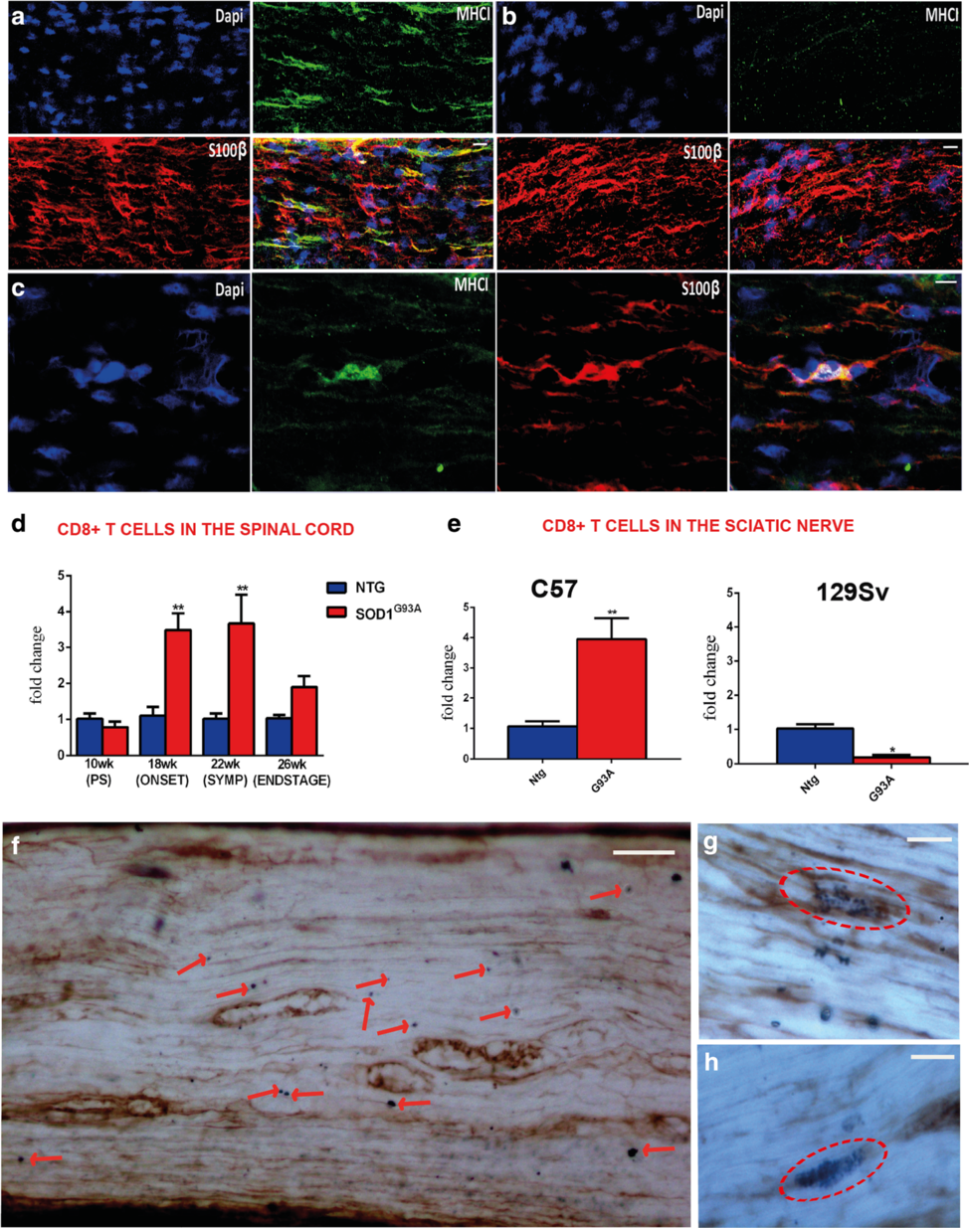
The greater expression of MHCI in the sciatic nerve of C57SOD1^G93A^ mice leads to an increase in CD8^+^ T cell infiltration. **a**–**c** Confocal micrographs of longitudinal section of the sciatic nerve of the **a** C57SOD1^G93A^ and **b** 129SvOD1^G93A^ mice showing greater activation of MHCI (*green*) and **c** its co-localization with SCs (*red*) in the peripheral nerve of the less severe phenotype. CD8^+^ T cells specifically infiltrate the spinal cord, sciatic nerve, and hind limb muscles of C57SOD1^G93A^ mice at disease onset. **d** Longitudinal real-time PCR for CD8 co-receptor transcript in the lumbar spinal cord of C57SOD1^G93A^ mice compared to Ntg littermates. Data are normalized to β-actin and expressed as the mean (±SEM)-fold change ratio between *n* = 5 C57SOD1^G93A^ and controls. **P value <0.01 by one-way ANOVA with Tukey’s post-analysis. **e** Real-time PCR for CD8 co-receptor transcript in the sciatic nerve of C57SOD1^G93A^ and 129SvSOD1^G93A^ mice compared to respective Ntg littermates at the symptom onset. Data are normalized to β-actin and expressed as the mean (±SEM)-fold change ratio between *n* = 5 C57SOD1^G93A^, *n* = 4 129SvSOD1^G93A^, and relative controls at disease onset. *P value <0.05; **P value <0.01 by Mann and Whitney test. **f** CD8^+^ T cells infiltrate the sciatic nerve of C57SDO1^G93A^ mice in close proximity to degenerating motor axons expressing high levels of MHCI; (*blue*, CD8^+^ cells indicated by *red arrows*; brown, MHCI); (*scale bar*, 50 μm). **g**, **h** High magnification highlights CD8^+^ cell clusters wrapping MHCI^**+**^ motor axons (*scale bar* 20 μm)

Under basal conditions, the levels of MHCI were very low in the lumbar spinal cord MN perikarya (Additional file 1: Figure S3c), while the protein was highly expressed in microglia and oligodendrocytes surrounding MNs [14] (Additional file 1: Figure S3d) and in efferent motor axons (Additional file 1: Figure S3e). β2M and LMP7 were upregulated and colocalized within a sub-population of MN perikarya in the C57SOD1^G93A^ mice but not in the control littermates (*n* = 5) (Additional file 1: Figure S3f–h). However, like the MHCI, both LMP7 and β2M were more expressed in the sciatic nerve of the C57SOD1^G93A^ than the Ntg mice (Fig. 4a, b; Additional file 1: Figure S4a, b) and colocalized in motor axons with SMI-31, a specific axonal marker (Fig. 4c–e; Additional file 1: Figure S4c–e). LMP7 and MHCI intensely colocalized in swollen and vacuolated regions of the axons reminiscent of Wallerian axonal degeneration (Fig. 4f–i). High levels of LMP7 were also observed in obturator nerve biopsies from a sporadic ALS patient (Fig. 4j–r), suggesting that activation of this pathway is not confined to SOD1-related ALS. MHCI [14] and β2M, but not LMP7, were also highly expressed at NMJs in C57SOD1^G93A^ mice (Fig. 4s, v; Additional file 1: Figure S4f). In addition to motor axons, MHCI was more expressed in SCs of C57SOD1^G93A^ mice than 129Sv^G93A^ mice (Fig. 5a–c). Since MHCI presentation is critical for adaptive immunity, we examined CD8^+^ T cell infiltration in the spinal cord and sciatic nerves of ALS mice. The transcript for the CD8^+^ T cell receptor was highly overexpressed in the spinal cords of the C57SOD1^G93A^ mice at disease onset and the symptomatic stage (Fig. 5d) mimicking the pattern of expression of *MhcI* and *Lmp7* mRNAs in laser-captured MNs (Additional file 1: Figure S3b). In contrast, there was no significant change in CD8^+^ T cell receptors within the spinal cords of 129SvG93A mice compared to the Ntg129Sv mice at disease onset (Additional file 1: Figure S5). CD8 mRNA levels were also higher in the sciatic nerve of C57SOD1^G93A^ mice than the Ntg littermates at disease onset (Fig. 5e). This was further confirmed by immunohistochemistry, showing CD8^+^ T lymphocyte infiltration in the sciatic nerves of the C57SOD1^G93A^ mice (Fig. 5f), forming clusters close to degenerating motor axons expressing high levels of MHCI (Fig. 5g, h). In contrast, CD8 mRNA was downregulated in the sciatic nerve of the 129SvSOD1^G93A^ mice at disease onset compared to the Ntg littermates (Fig. 5e).

### Peripheral activation of CCL2 and complement subunit C3 is responsible for greater macrophage recruitment in the sciatic nerve of slow-progressing mice

**Fig. 6:**
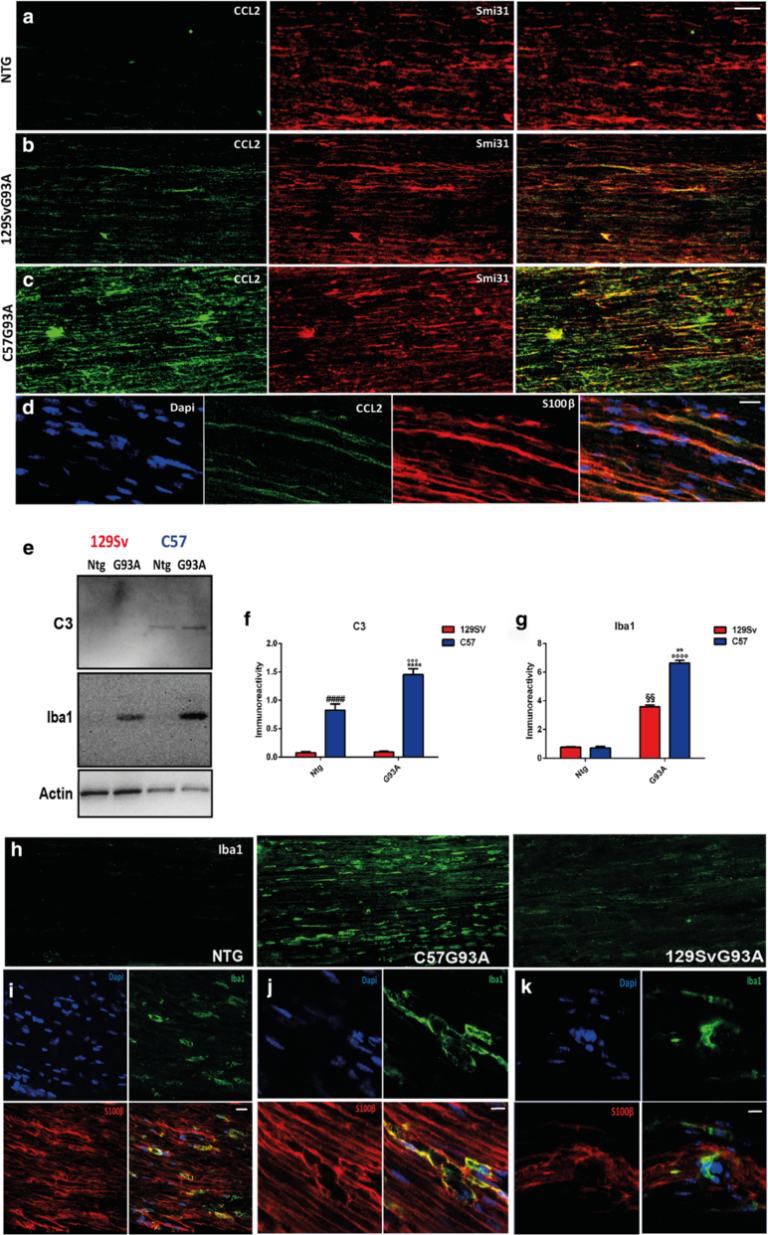
The C57SOD1^G93A^ mice have greater macrophagic activity than the 129SvSOD1^G93A^ in the sciatic nerve at disease onset. **a–c** Confocal micrographs show higher levels of CCL2 (*green*) in the sciatic nerve of C57SOD1^G93A^ than 129SvSOD1^G93A^ at disease onset and co-localization with the axonal marker Smi31 (*red*); *scale bars*, 50 μm. **d** Confocal micrographs showing the co-localization of the signal between CCL2 (*green*) and S100β (*red*) in the sciatic nerve of C57SOD1^G93A^ mice at disease onset; nuclei were stained with Dapi (*blue*); *scale bar*, 20 μm. **e** Representative western blot images of Iba1 and C3 on sciatic nerve extracts from the C57SOD1^G93A^, 129SvSOD1^G93A^ mice, and Ntg littermates at disease onset. Densitometric analysis indicates a correlation between the levels of activation of **f** C3 and the marked upregulation of **g** Iba1 expression in the C57SOD1^G93A^ mice. The graph represents the mean ± SEM (*n* = 4 mice per group). ****P value *<*0.0001; **P value <0.01 (C57G93A vs 129SvG93A); °°°°*P* value <0.0001; °°°*P* value <0.005 (C57G93A vs 129SvNtg and or C57Ntg); ^§§^ *P* value <0.01 (129SvG93A vs Ntg 129SvNtg and C57Ntg); ^####^ *P* value *<*0.0001 (C57Ntg vs 129SvNtg and 129SvG93A) by two-way ANOVA with Tukey’s post-analysis. **h** Confocal micrographs showing higher levels of Iba1 (*green*) in the sciatic nerve of C57SOD1^G93A^ at disease onset; *scale bar*, 50 μm. **i, j** Confocal micrographs of longitudinal section of sciatic nerve of the C57SOD1^G93A^ mice showing the clear co-localization of signals between S100beta (*red*) and Iba1 (*green*); nuclei were stained with Dapi (*blue*); *scale bars*, (i) 20 μm; (j) 10 μm. **k** Confocal micrographs of longitudinal section of sciatic nerve of the C57SOD1 ^G93A^ mice showing the presence of Iba^**+**^ (*green*) S100β^**−**^ (*red*) cells; nuclei were stained with Dapi (*blue*); *scale bar*, 10 μm

Besides MHCI, we previously showed that CCL2 and C3 are specifically upregulated by MNs in slow-progressing mice at disease onset [14]. In this study, there was differential expression of the two proteins in the peripheral nerves of the two mouse strains as well. The levels of CCL2 were upregulated in the sciatic nerve of C57SOD1^G93A^ mice whereas they were almost absent in 129SvSOD1^G93A^ mice (Fig. 6a–f). Increased CCL2 immunostaining colocalized with SMI31 in motor axons (Fig. 6c) and with S100β in SCs (Fig. 6d) of slow-progressing mice. Since CCL2 is a monocyte/macrophage chemoattractant protein, we measured the levels and the distribution of the macrophage-specific protein Iba1 in the sciatic nerves of the two mouse strains. Immunoblot and immunohistochemistry both showed higher levels of Iba1 in the sciatic nerves of the C57SOD1^G93A^ than in the 129SvSOD1^G93A^ mice at disease onset (Fig. 6e, g, h). Iba1 was expressed not only by macrophages (Fig. 6i–k) but also by SCs (Fig. 6i, j; Additional file 1: Figure S5a) in the sciatic nerve of the C57SOD1^G93A^ mice. The levels of complement C3 were also significantly raised in the sciatic nerve of the C57SOD1^G93A^ but not 129SvSOD1^G93A^ mice (Fig. 6e, f). The activation of the complement is also responsible for the recruitment of blood macrophages in SOD1^G93A^ mice [5]. Despite such marked recruitment of macrophages, the RT-qPCR on the sciatic nerve extracts showed no difference in the expression levels of pro-inflammatory factors such as TNFα, IL6, and Ly6c; however, the M2-myeloid marker Ym1 was higher in the C57SOD1^G93A^ mice than the Ntg mice (Additional file 1: Figure S6b–e).

## Discussion

This study demonstrates that the integrity of PNS plays an essential role in governing the onset of the disease and the speed of its progression in two mouse models of familial ALS carrying the same amount of human SOD1^G93A^ transgene on a different genetic background and suggests that this phenomenon is influenced by the immune system. In fact, while both mouse ALS strains present similar loss of MNs during the disease course [13], the rapid-progressor 129SvSOD1^G93A^ mice present earlier axonal impairment and denervation of hind limb muscles as indicated by the marked upregulation of the AChRγ-subunit mRNA levels in the TA muscles compared to the C57SOD1^G93A^ mice. Normally, the AChRγ-subunit it is almost undetectable or at very low levels in innervated, normally active muscle, while it becomes abundant along the whole fiber length in denervated muscle or in muscle in which the neuromuscular contact is intact but the release of transmitter is blocked [22]. This is in line with the hypothesis that alterations at the NMJ might precede the massive MN loss [2]. Muscle denervation is associated with a with marked disruption of motor axons in 129SvSOD1^G93A^ mice demonstrated by the reduced levels of acetylated tubulin and neurofilaments, two indices of axonal transport and structure [24, 25] and by the increase of β-importin, a marker of axonal stress [23] in their sciatic nerve.

The extent of the de-differentiation and proliferation of SCs during regeneration of the damaged motor axons in SOD1^G93A^ mice also seems to play a relevant role modulation the disease course in these ALS mouse models. In fact, the basal expression in sciatic nerves of P-ERK, which is fundamental to stimulate SCs de-differentiation during reinnervation [26], is much lower in the 129Sv NTg than in the C57 NTg mice. This difference was reflected in a reduced level of activation in 129SvSOD1^G93A^ mice in comparison to C57SOD1^G93A^ mice. The same difference is evident for the expression of GFAP in the sciatic nerves, which is essential to promote the proliferation of SCs after damage [27, 30]. In fact, depletion of this protein in the sciatic nerves has been reported to delay nerve regeneration after axotomy in mice [27]. RP-CARS structural analysis of the explanted sciatic nerves further supports this concept at morphological level showing significant differences in myelin anisotropy between the C57 and 129sv background. Statistically significant differences were also found in the *β* value between the two strains (and, in particular, between SOD1G93A mice) highlighting the contribution of mouse genetic background in promoting the integrity of the myelin structural architecture during disease progression.

This result is in accordance with the studies by Bieber et al. [31] who, by using different inbred mouse strains model of chronic progressive multiple sclerosis, clearly demonstrated that the axonal integrity and neurologic function is mainly affected by genetic background-related loci inherited as dominant traits. In parallel, the study of different experimental mouse models of axon remyelination has allowed identification of potential genetic factors able to enhance axonal remyelination [32]. For instance, fibroblast growth factor 2 (FGF2) and platelet-derived growth factor (PDGF) promote the proliferation of oligodendrocyte precursor cells (OPCs) [33, 34], while insulin growth factor 1 (IGF1) stimulates both OPCs differentiation and axon myelination [35]. In addition, P-ERK, which we observed to be more expressed in the C57 than the 129Sv mice, is essential to coordinate the stress response of myelinating oligodendrocytes following insult [36].

The mouse genetic background also profoundly influences the immune system response. In fact, inflammatory cell recruitment is markedly lower in the 129Sv than in the C57 mice following thioglycolate intraperitoneal injection [37]. This is consistent with our observation of a reduced macrophage and T cell infiltration in the peripheral nerves of the 129SvSOD1^G93A^ compared to C57SOD1^G93A^ mice. The striking activation of the immunoproteasome and MHCI in motor axons and SCs of C57SOD1^G93A^ mice correlates with the specific infiltration of CD8^+^ T cells in sciatic nerves. The recruitment of macrophages is related to the coordinated and specific activation of both CCL2 and the complement C3 subunit. Apparently, this reactive immune response is not accompanied by inflammation as no significant upregulation of pro-inflammatory factors (TNFα; IL-6; Ly6c) were observed in the sciatic nerves of the C57SOD1^G93A^ mice. By contrast, an increase in the M2 marker, Ym1, suggested a shift towards a protective macrophage phenotype. Thus, these findings indicate a beneficial role of immune cell infiltrates during the disease progression of SOD1-related ALS mice.

Several observations suggest the importance of immune infiltration in CNS repair [38–42]. For instance, recent studies demonstrated a beneficial role of macrophage infiltration in mouse models of experimental autoimmune encephalitis (EAE). In this regard, the transfer of M2-polarized monocytes resulted in a suppression of EAE [43, 44]. Additional studies in EAE mouse models and other disorders indicate that T cells responses can facilitate CNS repair at a later stage by restricting a prominent secondary wave of damage [45–48].

**Fig. 7:**
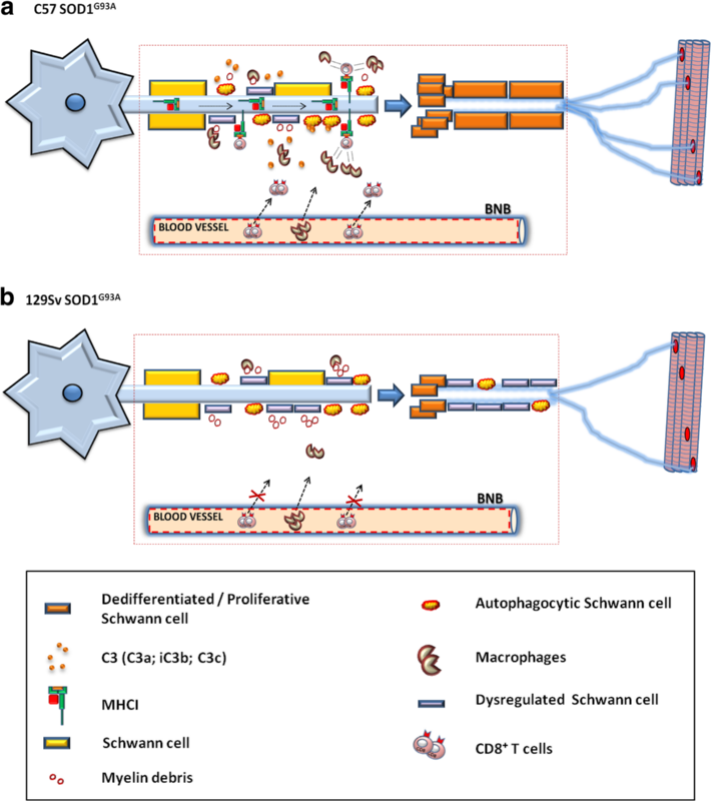
Immune cell infiltration in the PNS of transgenic ALS mice promotes Schwann cells (SCs) proliferation and axonal maintenance. Schematic representation of the PNS of C57SOD1^G93A^ mice and 129SvSOD1^G93A^ mice during disease progression. **a** In the C57SOD1 ^G93A^ mice, the activation of MHCI, autophagocytic SCs, and C3 byproducts leads to the recruitment and interaction of CD8^+^ T cells and macrophages at the site of injury. CD8^+^ T cells and macrophage coordinated activity potentiates the rate of degradation of defective SCs (and myelin debris) in order to stimulate the de-differentiation and proliferation of SCs and, finally, the remyelination of motor axons and NMJs. **b** In the 129SvSOD1^G93A^ mice, the lower phagocytic activity of SCs/macrophage and the lack of CD8^+^ T cell infiltration result in less removal of defective SCs in addition to reduced SCs de-differentiation/proliferation which lead to earlier muscle denervation

Based on these and our findings, we hypothesize that in the PNS of slow-progressing SOD1 mutant mice the SCs together with CD8^+^ T cells and peripheral macrophages cooperate in the targeted destruction of defective motor fibers expressing axon growth inhibitors (e.g., myelin debris). This would then promote the de-differentiation and proliferation of SCs to create a growth-permissive milieu for new neurites generated following axonal injury [49–51] (Fig. 7a, b).

This scenario resembles that previously reported in experimental mouse models of axon remyelination in which the proliferation and differentiation of oligodendrocytes precursor cells were accompanied by immune cells infiltration [52–54]. Notably, the depletion or pharmacological inhibition of macrophages following toxin- or virus-induced demyelination leads to an impairment of remyelination [55–57], as does an absence of T cells [58, 59]. This evidence suggests that protective autoimmunity is a physiologically evoked mechanism through which the body harnesses the immune system in order to protect itself against neuronal degeneration.

This mechanism is more prominent in the PNS where immune cell infiltration is not restricted, in contrast to the CNS [38]. Barrette et al. recently demonstrated that peripheral macrophages were essential to promote the production of neurotrophins (NT-3; BDNF) in regenerating motor axons and macrophage depletion significantly slowed axon regeneration and functional recovery after sciatic nerve injury [60]. Moreover, the CCL2-mediated neuron-macrophage interaction is vital for amplification and maintenance of enhanced regenerative capacity by preconditioning peripheral nerve injury [61]. In vivo imaging in a SOD1^G93A^ mice revealed distinct inflammatory activity of CNS microglia compared to PNS macrophages, the latter showing no substantial morphological reaction during degeneration of peripheral motor axons, but a passive role in clearing debris, particularly lipid-rich myelin [62]. In keeping with this, myelin ingestion by myeloid cells in vitro induces a foamy appearance and confers an anti-inflammatory function [63]. This evidence indicates about an essential role of hematogenous macrophages in promoting the remyelination of defective motor fibers. In addition, CD8^+^ T cells infiltrating the peripheral nerves may be essential not only to promote the phagocytosis of myelin debris but also for limiting the activity of effector T cells and macrophages once the remyelination is completed (recovery phase) [49]. In fact, it is well known that the CD8^+^ T cells include a subset of regulatory T cells (CD8^+^ Tregs) expressing specific regulatory markers such as CD103, FoxP3, or CD122 [64]. These were the first described subset of T cells capable of inducing immune suppression in vitro [65] and showing a greater immunosuppressive effect than CD4^+^ Tregs in a number of models including EAE [66, 67].

## Conclusions

It is generally accepted that terminal axonal degeneration and NMJ denervation occur before the loss of MNs in ALS [2]. However, this evidence was largely neglected for years with the result that no preclinical trials designed to inhibit degeneration of MN cell bodies in SOD1^G93A^ mice yielded the expected outcomes in terms of therapeutic benefit. There is growing evidence now that even complete rescue of MN somata may not be enough to counteract the disease progression in SOD1^G93A^mice [68]. This agrees with our findings which highlight that the extent of axonal and neuromuscular dysfunction, rather than MNs loss, seems predictive of a more aggressive phenotype. The level of degeneration (or preservation) of this compartment may therefore underlie the heterogeneity of ALS, which is a powerful confounding factor in evaluating the real efficacy of treatments [7].

Our results demonstrate that genetic background influences the basal levels of proteins that are essential to better preserve the myelination of peripheral motor axons during disease progression. In addition, we found that the maintenance of PNS function through the interaction with a specific immune response is essential for delaying disease progression in transgenic mouse models suggesting that genetic differences in immune responsiveness could affect the outcome of ALS. Accordingly, it will be extremely relevant to conduct genetic linkage analysis on F1 backcross generation between fast and slow mouse strains to search for genetic loci linked to enhanced remyelination and immune activation.

With respect to the translation of these findings to human ALS, so far there are no studies comparing the PNS of fast- and slow-progressing ALS patients due to the limited availability of tissues. However, here we demonstrate for the first time that one of the main components of the MHCI pathway, the LMP7, is upregulated in the motor axon of a sporadic ALS patient indicating that this phenomenon is not strictly related to the overexpression of mutant SOD1. We have not been able to investigate whether this variation correlates with a protective or detrimental effect on motor axons. Nevertheless, the failure of anti-inflammatory and anti-immune therapies in clinical trials [69] highlights the incomplete knowledge of the inappropriate knowledge of the dynamic immunological changes that occur during the disease progression and indirectly supports a reconsideration of the immune system in ALS.

The SOD1^G93A^ mouse used in this study is representative of a low percentage of ALS patients [1]. However, so far it is the only model that well recapitulates the clinical and pathological characteristics of the human disease [1]. For this reason, we expect that a better understanding of the molecular mechanisms of cross-talk between damaged axon signaling and the immune response in SOD1^G93A^ mice should help in finding new approaches for promoting axonal regeneration and muscle reinnervation in ALS. In parallel, the discovery of genetic factors that may be important for slowing disease progression could allow identification of molecular signatures as potential druggable targets and disease biomarkers for homogeneous subsets of ALS patients.

## Additional file

Additional file 1: Figure S1. Normalized frequency histograms of the α values computed from all the acquired RP-CARS z-stacks in the four conditions: Ntg vs. SOD1G93A and C57 vs. 129Sv. **Figure S2.** GFAP and P-ERK expression is reduced in the sciatic nerve of 129Sv_Ntg. **Figure S3.** H2-K_D (MhcI; red), and β2m (blue) mRNA levels from laser captured MNs of C57Ntg versus 129SvNtg mice. H2-K_D (red), Lmp7 (green) and β2m (blue) and Tap1 (black) longitudinal mRNA levels from laser captured MNs of C57SOD1G93A versus Ntg littermates during the progression of the disease. MHCI immunohistochemistry shows an increased and widely diffuse expression in the ventral grey and white matter of the lumbar spinal cord of C57SOD1G93A mice at disease onset compared to Ntg littermates β2M (green) and LMP7 (red) colocalization in a subset of lumbar spinal MNs (blue, NT) of C57SOD1G93A mice (inset) at the onset compared to Ntg littermates (scale bar: 50 μm; inset scale bar: 20 μm). Representative western blot of LMP7 and β2M expression in protein extracts from longitudinally dissected lumbar ventral spinal cord of C57SOD1G93A mice at the disease onset versus Ntg littermates. **Figure S4.** β2M is activated in the peripheral nerves of SOD1G93A mice. **Figure S5.** Real-time PCR for CD8+ co-receptor transcript in the spinal cord of 129SvSOD1G93A mice and Ntg littermates at disease onset. **Figure S6.** Confocal Z-stack orthogonal projection of a longitudinal section of sciatic nerve of C57SOD1G93A mice showing the co-localization of signals between S100beta (red) and Iba1(green), dapi (blue). RT-qPCR for Ym1, Tnfα, Il6 and Ly6c co-receptor transcript in the sciatic nerve of C57SOD1G93A mice compared to Ntg littermates at disease onset. (PDF 1222 kb)

## Abbreviations

ALS: Amyotrophic lateral sclerosis
CCL2: Chemokine (C-C motif) ligand 2
LMP7: Low molecular mass 7
MHCI: Major histocompatibility complex I
MN: Motor neuron
NMJ: Neuromuscular junction
Ntg: Non-transgenic
PNS: Peripheral nervous system
SC: Schwann cell
SOD1: Cu/Zn superoxide dismutase
TAP1: Transporter of antigen peptide 1
