# Supplementary material for: Immune response in peripheral axons delays disease progression in SOD1G93A mice
Source: J Neuroinflammation. 2016 Oct 7;13:261. doi: 10.1186/s12974-016-0732-2 (PMC5055725; doi:10.1186/s12974-016-0732-2)
Supplement: Additional file 1: Figure S1. — Normalized frequency histograms of the α values computed from all the acquired RP-CARS z-stacks in the four conditions: Ntg vs. SOD1G93A and C57 vs. 129Sv. Figure S2. GFAP and P-ERK expression is reduced in the sciatic nerve of 129Sv_Ntg. Figure S3. H2-K_D (MhcI; red), and β2m (blue) mRNA levels from laser captured MNs of C57Ntg versus 129SvNtg mice. H2-K_D (red), Lmp7 (green) and β2m (blue) and Tap1 (black) longitudinal mRNA levels from laser captured MNs of C57SOD1G93A versus Ntg littermates during the progression of the disease. MHCI immunohistochemistry shows an increased and widely diffuse expression in the ventral grey and white matter of the lumbar spinal cord of C57SOD1G93A mice at disease onset compared to Ntg littermates β2M (green) and LMP7 (red) colocalization in a subset of lumbar spinal MNs (blue, NT) of C57SOD1G93A mice (inset) at the onset compared to Ntg littermates (scale bar: 50 μm; inset scale bar: 20 μm). Representative western blot of LMP7 and β2M expression in protein extracts from longitudinally dissected lumbar ventral spinal cord of C57SOD1G93A mice at the disease onset versus Ntg littermates. Figure S4. β2M is activated in the peripheral nerves of SOD1G93A mice. Figure S5. Real-time PCR for CD8+ co-receptor transcript in the spinal cord of 129SvSOD1G93A mice and Ntg littermates at disease onset. Figure S6. Confocal Z-stack orthogonal projection of a longitudinal section of sciatic nerve of C57SOD1G93A mice showing the co-localization of signals between S100beta (red) and Iba1(green), dapi (blue). RT-qPCR for Ym1, Tnfα, Il6 and Ly6c co-receptor transcript in the sciatic nerve of C57SOD1G93A mice compared to Ntg littermates at disease onset. (PDF 1222 kb) [file 12974_2016_732_MOESM1_ESM.pdf]

Immune response in peripheral axons delays disease progression in SOD1^{G93A} mice

Journal of Neuroinflammation

Giovanni Nardo[1*+], Maria Chiara Trolese[1*], Giuseppe de Vito,[2,3], Roberta Cecchi[2], Nilo Riva[4], Giorgia Dina[3], Paul R. Heath[5], Angelo Quattrini[4], Pamela J. Shaw[5], Vincenzo Piazza[3], Caterina Bendotti[1+]

Microarray data

The data discussed here have been deposited in the NCBI Gene Expression Omnibus and are accessible through GEO Series accession number GSE46298.

Western Blot

Membranes were immunoblotted with the following primary antibodies: rabbit polyclonal anti-Lmp7 (1:2000; AbD Serotec); rabbit polyclonal anti-β2M (1:1000; Proteintech); mouse anti β-actin (1:30000; Chemicon); mouse anti importin-β; (1:5000; Millipore); mouse anti-acetylated tubulin (1:1000; Millipore); mouse anti β-tubulin (1:1000; Millipore); rabbit anti-ERK (K23, 1:1000; Santa Cruz Biotech); mouse anti-phospho-ERK (E4, 1:1000; Santa Cruz Biotech); rabbit anti-NF200 (1:1000; Sigma-Aldrich); mouse anti-GFAP (1:2000; Millipore); rabbit polyclonal anti-C3 (H300, 1:100; Santa Cruz) followed by HRP-conjugated secondary antibodies (Santa Cruz) and developed by Luminata Forte Western Chemiluminescent HRP Substrate (Millipore) on the Chemi-Doc XRS system (Bio-Rad).

Quantitative reverse transcription PCR

The following probes were used for the quantitative reverse transcription PCR: CD8 alpha receptor (Mm01182107_g1; Life technologies); Tumour Necrosis Factor alpha (TNFα; Mm00443258_m1; Life Technologies); Interleukin 6 (IL6; Mm00446190_m1; Life technologies); Chitinase-like 3 (Ym1; Mm00657889_mH; Life Technologies); Lymphocytes antigen 6 complex (Ly6c; Mm00841873_m1; Life Technologies) and CHRNG (Cholinergic receptor, nicotinic, gamma subunit; Mm00437419_m1; Life Technologies); β-actin (4310881E; Life Technologies).

CARS acquisition and data analysis

The CARS contrast-generation mechanism is based on the chemical properties the sample: a couple of incoming photons ("pump" and "Stokes") resonantly and coherently excite the vibrational modes of a molecular bond of interest (the symmetric and antisymmetric stretch modes of CH_2 bonds, that are particularly abundant in the myelin acyl chains in the case here of interest), a third photon ("pump" again, in this case) is used to de-excite the molecular vibration, thus emitting a fourth photon, i. e. the CARS signal [1].

The RP-CARS microscopy is a recent development [2] of the original CARS method. Here, a circularly polarized Stokes beam and a linearly polarized pump beam (with a rotating polarization plane) are exploited to extract in real time the average in-plane orientation anisotropy of the molecular bonds (α) and their average orientation (φ) within the sub-micrometric excitation volume. The setup employed and the specific experimental implementation are described in great details in de Vito et al. [3]. The α-value data are analyzed as described in [4] and in the main text.

The φ-value analysis, performed with a custom-made Python software, is detailed in [5] and, for the sake of clarity, is described here briefly. The pixels associated to the myelin walls are selected by means of a threshold value (set as the stack-based 90th percentile of the RP-CARS signal averaged over the pump-beam polarization-plane angle), followed by the binary closure of the mask. We define a spatial scale by partitioning the thresholded images of each z-stacks in square sub-images of a given size. The resultant length [5], defined as: $\left|\sum_{j=1}^{N} exp\left(i\phi_j\right)/N\right|^2$, is computed for the φ values of the (N) thresholded pixels in each sub-image. Finally, the software computes from all the sub-images of a z-stack a single indicator (called β-value), by averaging the resultant lengths using the number of thresholded pixels present in each sub-image as weights

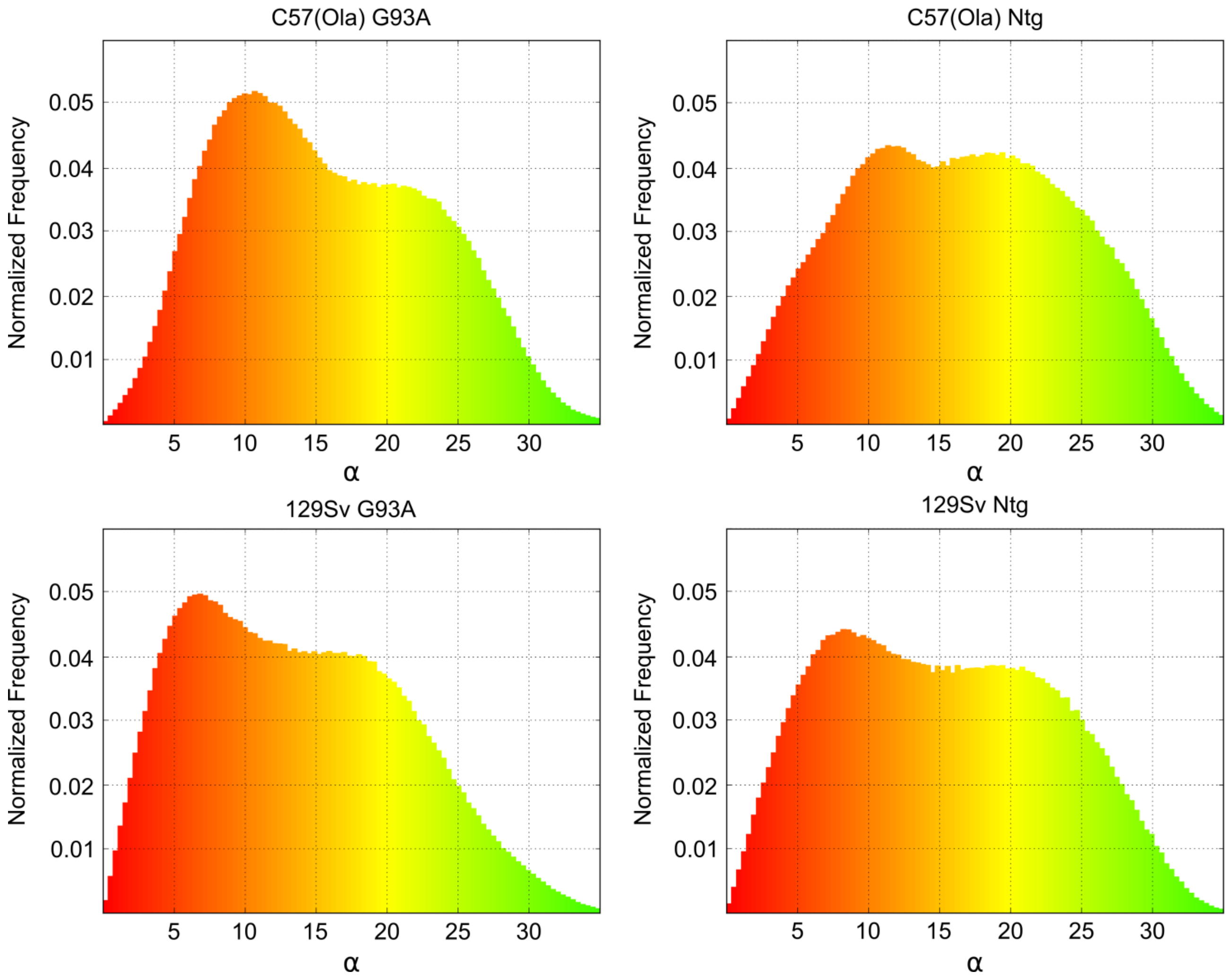

Additional Fig. 1: Normalized frequency histograms of the α values computed from all the acquired RP-CARS z-stacks in the four conditions: Ntg vs. $SOD1^{G93A}$ and C57 vs. 129Sv. The z-stacks were thresholded to select only the myelin-associated pixel, as described in [3] and in the main text. The histograms are color-coded according to the α value as in Figure 3b

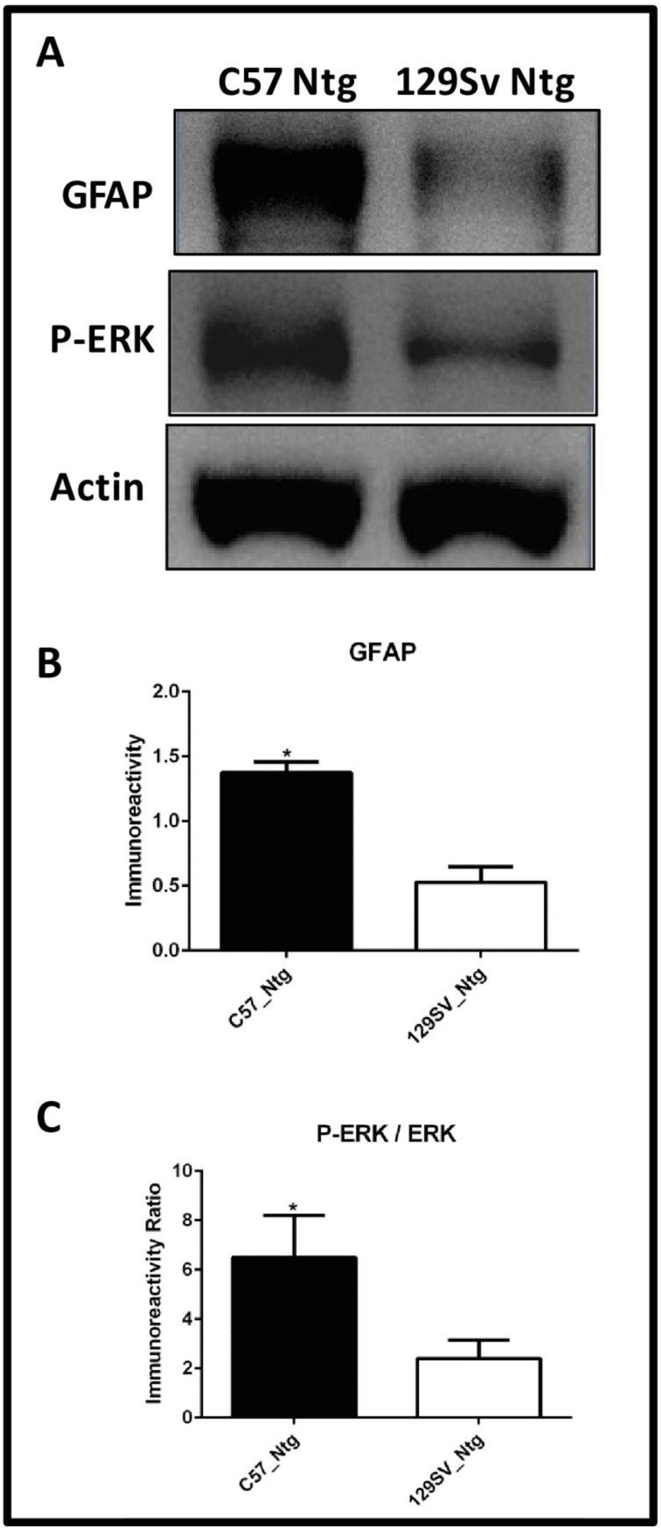

Additional Fig. 2 GFAP and P-ERK expression is reduced in the sciatic nerve of 129Sv_Ntg. (**a**) Representative immunoblot images of P-ERK and GFAP in the sciatic nerve extracts from 129Sv_Ntg, C57_Ntg at 98 d. (**b, c**) Densitometric analysis revealed a remarkable reduction of GFAP and phospho-ERK in age matched Ntg+/+, Ntg-/- mice. Data are reported as mean ± SEM (n = 4 mice). **P-value < 0.05* by Mann-Whitney's test

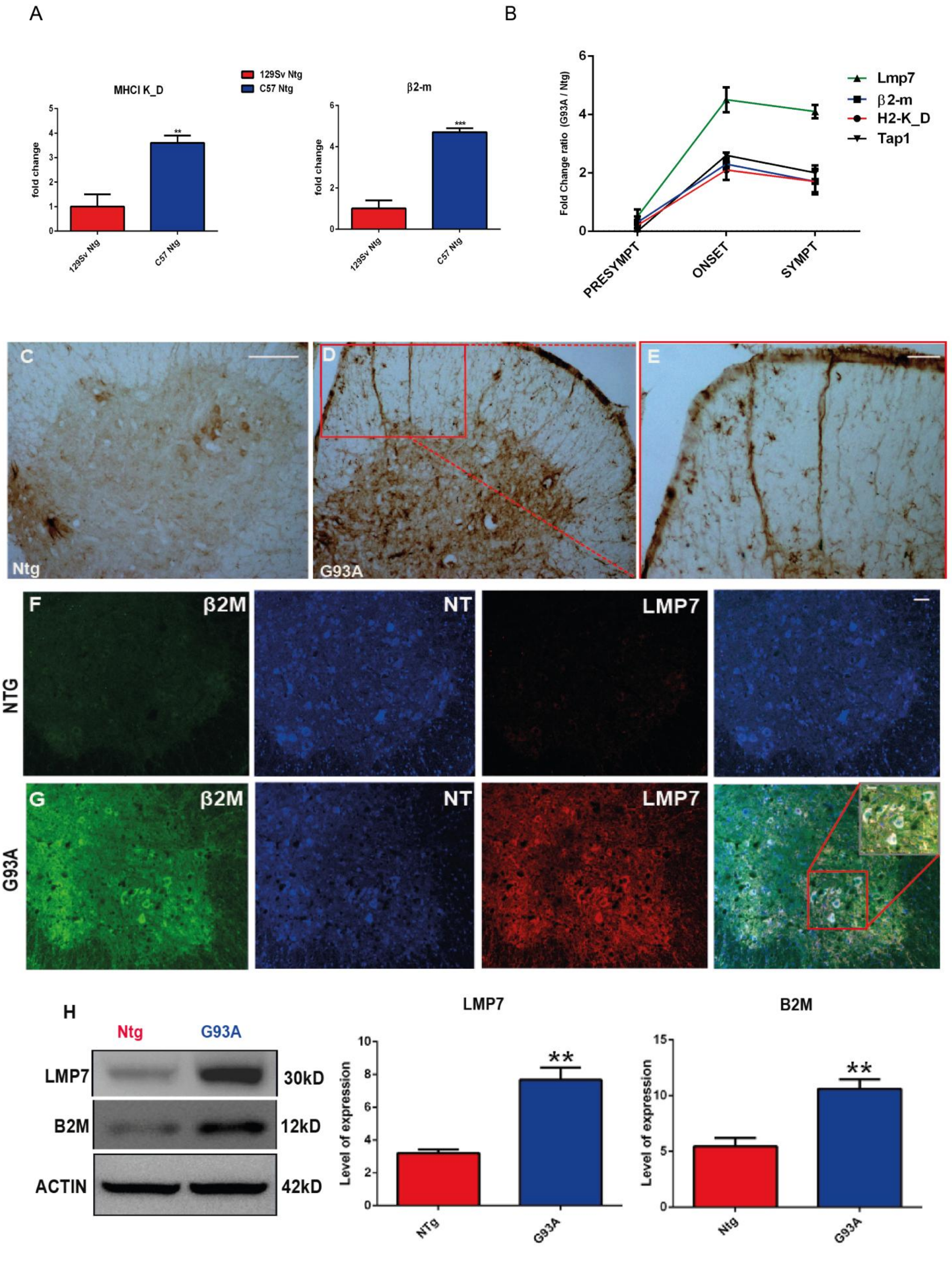
A
B
MHCI K_D
129Sv Ntg
C57 Ntg
β2-m
fold change
Fold Change ratio (G93A / Ntg)
Lmp7
β2-m
H2-K_D
Tap1
PRESYMPT
ONSET
SYMPT
C
D
E
Ntg
G93A
F
G
β2M
NT
LMP7
NTG
G93A
H
Ntg
G93A
LMP7
B2M
ACTIN
30kD
12kD
42kD
LMP7
B2M
Level of expression
NTg
G93A
Ntg
G93A

Additional Fig. 3. a *H2-K_D* (*MhcI*; red), and *β2m* (blue) mRNA levels from laser captured MNs of C57Ntg versus 129SvNtg mice. mRNA levels are expressed as mean fold change ratio (± SEM) between C57Ntg (n = 4) and 129SvNTg (n = 4) mice. **P-value < 0.01; ***P-value < 0.001 by Mann and Whitney's test. **b** *H2-K_D* (*red*), *Lmp7* (*green*) and *β2m* (*blue*) and *Tap1* (*black*) longitudinal mRNA levels from laser captured MNs of C57SOD1^{G93A} versus Ntg littermates during the progression of the disease. mRNA levels are expressed as mean fold change ratio (± SEM) between C57SOD1^{G93A} (n = 4) and NTg (n = 4) mice for each disease stage. Q-values ≤ 0.01. (**c-e**) MHCI immunohistochemistry shows an increased and widely diffuse expression in the ventral grey and white matter of the lumbar spinal cord of C57SOD1^{G93A} mice at disease onset (d) compared to Ntg littermates (c). Magnification of the white matter (e) shows ventral root efferent fibers intensely stained by MHCI; scale bars: (c, d) 100 µm, (e) 50um; (**f**, **g**) β2M (green) and LMP7 (red) colocalization in a subset of lumbar spinal MNs (blue, NT) of C57SOD1^{G93A} mice (g, inset) at the onset compared to (f) Ntg littermates (scale bar: 50 µm; inset scale bar: 20 µm). (**h**) Representative western blot of LMP7 and β2M expression in protein extracts from longitudinally dissected lumbar ventral spinal cord of C57SOD1^{G93A} mice at the disease onset versus Ntg littermates. Densitometric analysis of the bands indicates a marked upregulation of LMP7 and β2M expression in C57SOD1^{G93A} mice. Data are reported as mean ± SEM of Ntg = 4 and C57SOD1^{G93A} = 5 mice (Mann Whitney test ***P-value < 0.01*)

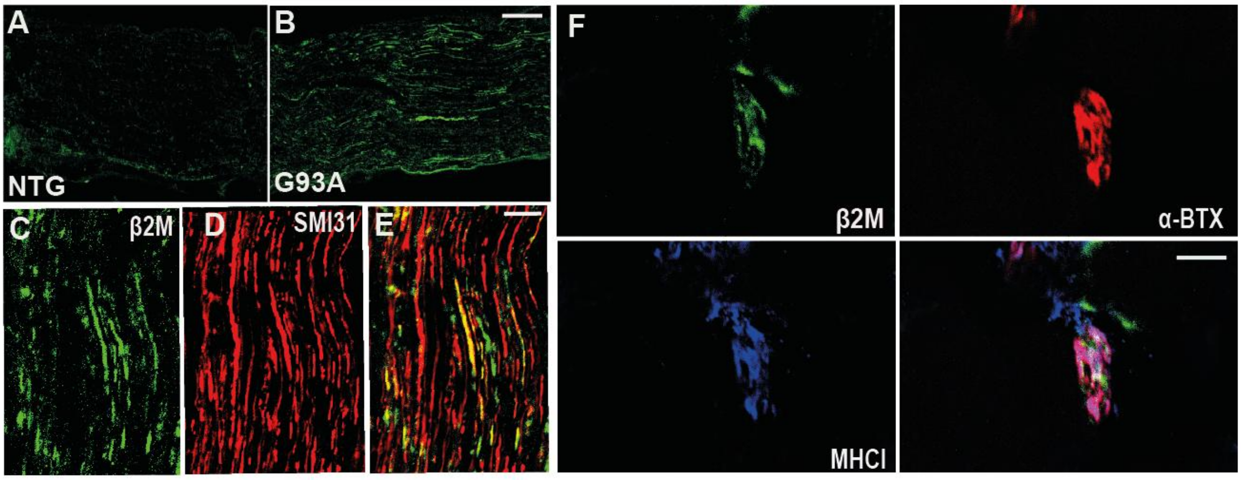

Additional Fig. 4 β2M is activated in the peripheral nerves of SOD1^{G93A} mice. (**a, b**) β2M immunoreactivity (green) shows a remarkable up-regulation in the sciatic nerve of C57SOD1^{G93A} mice at the disease onset (G93A) compared to Ntg littermate (NTG) (scale bar: 100μm) (**c-e**) Representative co-localisation of LMP7 with Smi31 (axonal marker; red) in the sciatic nerve of C57SOD1^{G93A} mice (scale bar: 20μm). (**f**) β2M is highly expressed at the level of the NMJ; β2M (green); α-BTX (red), MHCI (blue) (scale bar: 50 μm)

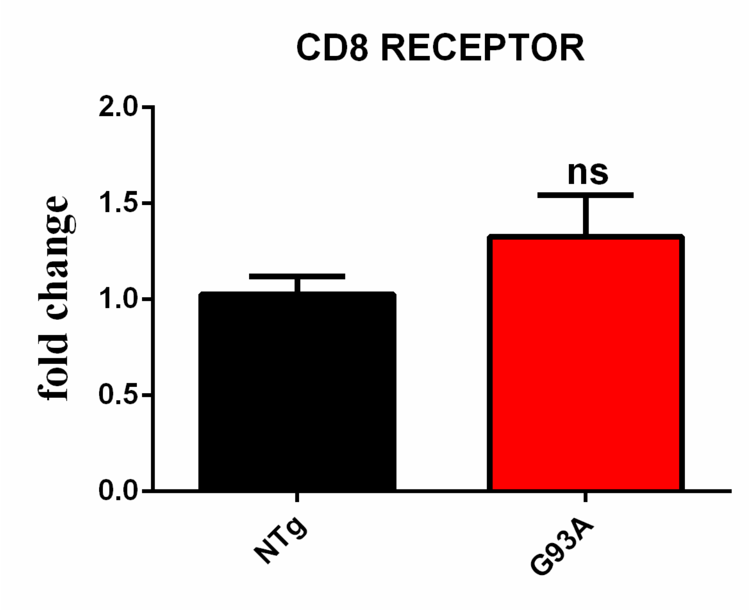

Additional Fig. 5 Real-time PCR for CD8^{+} co-receptor transcript in the spinal cord of 129SvSOD1^{G93A} mice and Ntg littermates at disease onset. Data are normalized to β-actin and expressed as the mean (± SEM) fold change ratio between n=4 129SvSOD1^{G93A} mice and relative controls. Ns by Mann and Whitney's test.

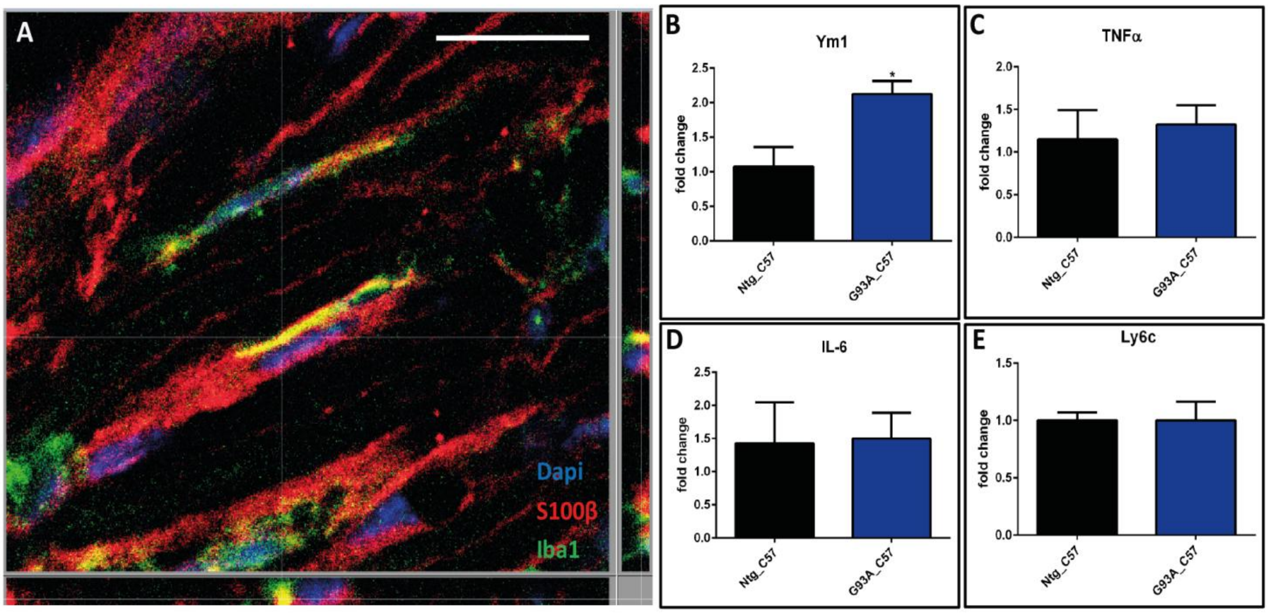

Additional Fig. 6 a Confocal Z-stack orthogonal projection of a longitudinal section of sciatic nerve of C57SOD1^{G93A} mice showing the co-localization of signals between S100beta (*red*) and Iba1(*green*), dapi (*blue*); *scale bars*: 20 µm (a); 10 µm. **b-e** RT-qPCR for (e) *Ym1*, (f) *Tnfα*, (g) *Il6* and (h) *Ly6c* co-receptor transcript in the sciatic nerve of C57SOD1^{G93A} mice compared to Ntg littermates at disease onset. Data are normalized to β-actin and expressed as the mean (± SEM) fold change ratio between n=4 C57SOD1^{G93A} and controls at disease onset. **P-value < 0.05* by Mann Whitney test.

References

1. Maker PD, Terhune RW **Study of Optical Effects Due to an Induced Polarization Third Order in the Electric Field Strength.** Phys. Rev. 1965; **137**: A801-A818.

2. de Vito G, Bifone A, Piazza V. **Rotating-polarization CARS microscopy: combining chemical and molecular orientation sensitivity.** Opt Express. 2012; **20**(28):29369-7

3. de Vito G, Cappello V, Tonazzini I, Cecchini M, Piazza V. **RP-CARS reveals molecular spatial order anomalies in myelin of an animal model of Krabbe disease.** J Biophotonics. 2016; DOI:10.1002/jbio.201500305.

4. Canta A, Chiorazzi A, Carozzi VA, Meregalli C, Oggioni N, Bossi M, Rodriguez-Menendez V, Avezza F, Crippa L, Lombardi R, et al: **Age-related changes in the function and structure of the peripheral sensory pathway in mice.** *Neurobiol Aging* 2016, **45:**136-148.

5. Mardia KV, Jupp PE **Directional Statistics**. Wiley 1999.
